# “Navigating Healthy Waters”: monitoring ship wastewater as a key defense against infectious diseases—a pilot study on a Mediterranean seaport

**DOI:** 10.3389/fpubh.2026.1786232

**Published:** 2026-04-23

**Authors:** Ileana Federigi, Nunzio Zotti, Marzia Pellegrini, Marco Verani, Alessandra Pagani, Alessandro Lattanzi, Alessandra Salvadori, Annalaura Carducci, Caterina Rizzo

**Affiliations:** 1 Hygiene and Environmental Virology Laboratory (HEVL), Department of Biology, University of Pisa, Pisa, Italy; 2 Department of Translational Research and New Medicine and Surgery Technologies, University of Pisa, Pisa, Italy; 3 USMAF-SASN Toscana Emilia-Romagna, Italian Ministry of Health, Livorno, Italy

**Keywords:** clinical surveillance, wastewater-based surveillance, sewage, cruise ship, SARS-CoV-2, enteric pathogens, fecal indicators, preparedness

## Abstract

Wastewater-based surveillance represents a non-invasive approach to monitor pathogen circulation, but data on cruise ships are scarce, although this enclosed environment is prone to infectious diseases spreading. We conducted a pilot study in a Mediterranean seaport (Italy) between July and October 2024, combining wastewater monitoring with onboard clinical surveillance. Twenty wastewater samples (10 untreated and 10 treated) were collected from 10 cruise ships. Viral detection targeted 10 human respiratory and gastrointestinal viruses. Bacterial and viral indicators (total coliform, *Escherichia coli*, intestinal enterococci, and somatic coliphages) were analyzed to evaluate wastewater treatment performance. Aggregated clinical data were extracted from medical reports and categorized using ICD-10 codes for symptoms of likely infectious viral origin. Norovirus genogroup II was consistently detected in untreated sewage (100%), followed by SARS-CoV-2 (60%), human adenovirus (30%), enterovirus, rotavirus, and hepatitis E virus (10–20%). Viral loads decreased in treated samples, although wastewater treatment efficiency varied widely among vessels: some ships exhibited a logarithmic abatement of less than 2 log_10_ units, with effluents showing microbiological concentrations above the thermotolerant coliform benchmark established for sewage treatment plant certification by the International Maritime Organization (IMO) guidelines. Clinical surveillance recorded more respiratory than gastrointestinal cases, with COVID-19 and influenza confirmed in several arrivals. The integration of environmental and clinical data provided complementary insights, particularly for SARS-CoV-2 detection in wastewater, which occurred in around 85% of ship arrivals with clinically diagnosed COVID-19 cases. These findings demonstrate that wastewater monitoring can complement clinical surveillance on cruise ships, by offering information on viral circulation. The variability observed in treatment efficacy underscores the need for harmonized standards and supports the inclusion of wastewater monitoring in maritime preparedness and resilience strategies to strengthen public health security against emerging infectious threats and enhance system adaptability to future outbreaks.

## 1 Introduction

Cruise tourism can raise some concerns about human health and environmental impacts. Cruise ships represent enclosed settings particularly prone to disease outbreaks, due to the semi-confined nature of life onboard and the use of common sources for food and water. These factors can facilitate the transmission of diarrheal diseases and other infections. Furthermore, the close interaction between large numbers of individuals increases the likelihood of exposure to infectious virus-laden respiratory secretions and the spread of an infection can be facilitated by the regular introduction of susceptible populations during the stop at the scheduled ports. For this reason, the construction and operation of ships need to respect health requirements, whose global reference is represented by the World Health Organization (WHO) guide to ship sanitation (1). The global purpose is to standardize the sanitary measures taken in ships, to safeguard the health of travelers and workers and to prevent the spread of infection from one country to another. This is achieved through the application of appropriate control measures, such as food safety controls, water safety plans, cleaning and disinfection procedures in air-conditioning systems, and comprehensive waste management. In addition, procedures, equipment, and dedicated facilities are provided to manage symptomatic individuals onboard. A number of specific guidance documents, conventions and regulations are also currently available. For example, in Europe, the European SHIPSAN Manual provides harmonized standards for the surveillance of communicable diseases on board (2), while the SHIPSAN TRAINET project proposes guidelines for outbreak management and training for port health workers (3). In the United States, the Vessel Sanitation Program (VSP) conducts ongoing surveillance of acute gastroenteritis and coordinates outbreak investigations on vessels (4). Nevertheless, waterborne and foodborne infectious diseases remain significant public health concerns on cruise ships. According to a WHO review of scientific literature, over 100 gastrointestinal outbreaks were reported globally in a 30-year period (1970–2003), affecting more than 16,000 individuals (5). This trend has continued in more recent years: in the United States alone, the CDC documented over 150 gastrointestinal outbreaks and more than 37,000 associated cases in just 15 years (2006–2019) based on data from the VSP surveillance system (6). Moreover, some bacterial gastrointestinal outbreaks have been associated with antibiotic-resistant strains, highlighting ships as critical settings for the spread of antimicrobial resistance (7). Ships are also vulnerable to respiratory infections, as clearly demonstrated during the COVID-19 pandemic. As an example, on February 2020, an outbreak of COVID-19 on the Diamond Princess cruise ship involved 712 cases, forcing approximately 3,700 passengers and crew to prolonged quarantine following arrival in Yokohama, Japan (8, 9).

Most of the human microbial pathogens circulating in the community are commonly released into feces, thus vessel sewages can contain microbiological pollution, whose uncontrolled discharge into the environment can pose a threat to human and environment health. Environmental impact of cruise ships is addressed by the International Maritime Organization (IMO), which adopted the International Convention for the Prevention of Pollution from Ships in 1973, later amended in 1978 named MARPOL 73/78 (10). The objective of the protocol is to manage the ship-related wastes, including sewage produced by the passengers and the crew, that need to be treated prior to discharge when the vessel is stationary or navigating within 3 to 12 nautical miles from the nearest land, as reported in the Annex IV of MARPOL 73/78. However, such an annex is not mandatory for signatory nations to MARPOL, moreover no sewage treatment is required for discharge at a distance greater than 12 miles. Hence, untreated or not adequately treated ship-originated sewage can contribute to the fecal microbial pollution of coastal environment, that is reported worldwide [e.g., (11–13)].

Therefore, there is growing attention to the role of ships as potential vehicles for the transboundary spread of pathogenic viruses, especially during pandemics. Outbreaks of H1N1 influenza and COVID-19 on various types of vessels (e.g., cruise, commercial, and cargo ships) have revealed considerable heterogeneity in the management of such events (14, 15). These experiences highlight the need to systematically integrate pandemic preparedness into maritime and port health protocols (16, 17). In this context, ship wastewater monitoring can be an effective and non-invasive tool for intercepting the spread of pathogenic viruses across maritime borders (18). In fact, wastewater surveillance has been already applied as valuable tool to understand the circulation of respiratory or enteric pathogens in other closed or semi-confined settings such as nursing homes, aircraft, and schools [e.g., (19–21)], despite critical issues related to analytical sensitivity and data variability, especially in case of respiratory virus detection (22, 23).

Despite the growing literature on respiratory and gastrointestinal outbreaks on cruise ships (24), the reported cases likely represent only a fraction of the total disease burden linked to ship-acquired infections, since the lack of a coordinated outbreak surveillance system (1). This surveillance gap also extends to the microbiological characterization of cruise ship wastewater, for which limited data are available, largely due to logistical challenges associated with sampling during active cruise operations (25).

Given the current lack of comprehensive data on infectious diseases on cruise ships, we conducted a pilot study aimed at studying circulation of viral pathogens on board through the integration of environmental data obtained from the microbiological monitoring of ship-originated sewages and epidemiological data from onboard clinical surveillance. Moreover, the performance of wastewater treatments towards microbial indicators and pathogens was also evaluated. This work contributes to strength health preparedness in the European maritime context and helps fill a critical gap in epidemiological surveillance within the maritime transport sector.

## 2 Materials and methods

### 2.1 Sampling stations and sewage samples

The study was conducted between July and October 2024 at a port on the Mediterranean, located in the north-west of Tuscany (Italy), where cruise ships operated by three different companies (hereafter referred to as Company A, Company B, and Company C) docked during the study period. Sampling was carried out at the time of docking for all participating companies, that provided access to their onboard wastewater treatment system for the collection of paired samples of influent (untreated) and effluent (treated) wastewaters (1 L each). Overall, 20 wastewater samples (10 untreated and 10 treated) were collected from 10 ships: five ships from Company A (10 samples), three ships from Company B (6 samples), and two ships from Company C (4 samples). For Companies A and C, sampling was performed on the same vessel from each company during multiple docking events, whereas, for Company B, sampling involved three different vessels, each sampled once during the study period. All samples were collected under aseptic conditions, transported under refrigeration to the Hygiene and Environmental Virology Laboratory (HEVL) – Department of Biology, University of Pisa, and processed within 24 h of collection.

### 2.2 Bacterial indicators

Bacterial indicators were quantified using IDEXX’s patented Defined Substrate Technology for the detection of total coliforms and *Escherichia coli* (Colilert-18), according to the technical standard BS EN ISO 9308-2 (26), and intestinal enterococci (Enterolert-E) following a method related to the standard BS EN ISO 7899-1 (27). Briefly, serial dilutions of each sample were prepared in sterile deionized water (undiluted, 1:10, 1:100, 1:1000), and 1 mL of each dilution was analyzed following the manufacturer’s instructions. Results were expressed as MPN (most probable number) per 100 mL sample.

### 2.3 Somatic coliphages

Somatic coliphages were determined using the Bluephage Enumeration of Somatic Coliphages Easy Kit for 1–10 mL samples, according to the technical standard BS EN ISO 10705-2:2001 (28), a double agar layer method with plaque detection on the *E. coli* WG5 host strain. Briefly, serial dilutions of each sample were prepared in sterile deionized water (undiluted, 1:10, 1:100, 1:1000), and 1 mL of each dilution was analyzed following the manufacturer’s instructions. Results were expressed as PFU (plaque forming units) per 100 mL sample.

### 2.4 Human pathogenic viruses

#### 2.4.1 Sample preparation and concentration

All wastewater samples were heat-inactivated at 56 °C for 30 min for biosafety purposes prior to concentration. Initially, six samples from three ship arrivals were processed using an ultrafiltration-based method. Briefly, after pre-filtration of the sample (1 L) through a membrane (130 cm^2^ surface area, 8 μm nominal porosity), the sample was concentrated by ultrafiltration through a 400 cm^2^ membrane with a 10 kDa molecular weight cut-off (Sartorius, Stenehouse, United Kingdom), following the protocol developed by HEVL (29). This method was discontinued due to technical challenges, primarily attributable to the high turbidity and density of the raw samples, which compromised the filtration process. The other 14 samples from seven ship arrivals were concentrated by centrifugation using polyethylene glycol (PEG) precipitation. In this procedure, the process control virus (Mengovirus strain vMC0) was added to the inactivated sample (45 mL) to monitor recovery efficiency. Briefly, the sample was centrifuged at 4500 × g for 30 min to remove solids, then the supernatant (40 mL) was mixed with PEG8000 and NaCl to precipitate viral particles using centrifugation at 12,000 × g for 2 h, as previously described [e.g., (23, 30, 31)]. The resulting pellet was resuspended in 2 mL of NucliSENS Lysis Buffer reagent (bioMerieux, Marcy l’Etoile, France) for nucleic acids extraction.

#### 2.4.2 Molecular analyses

Nucleic acids (DNA and RNA) were extracted with NucliSense EasyMag (bioMerieux, Marcy l’Etoile, France) based on magnetic silica beads, obtaining a final volume of 100 μL that has been further purified using One-Step PCR Inhibitor Removal Kit (Zymo Research, Irvine, CA, United States) and stored at −80 °C until molecular analysis. Real-time (RT)-qPCR assays were then performed for 10 viral targets: human adenovirus (HAdV), enterovirus, norovirus genogroup II (NoVggII), hepatitis A virus (HAV), hepatitis E virus (HEV), rotavirus, SARS-CoV-2, influenza A virus (IAV), and respiratory syncytial virus A and B (RSVA, RSVB). qPCR and RT-qPCR analyses were performed using TaqMan probe-based chemistry in 25 μL total reaction volumes, employing TaqMan Universal PCR Master Mix (Applied Biosystems – Thermo Fisher Scientific, Warrington, United Kingdom) for the detection of HAdV and AgPath-ID One-Step RT-PCR (Applied Biosystems - Thermo Fisher Scientific, Austin, TX, United States) for the detection of other viruses, respectively. Chemical and thermal protocols for each assay are detailed in Supplementary Table S1 and were previously used for the detection of respiratory and enteric viruses (23, 29, 32). For each target, viral titers were determined by preparing serial dilutions (from 1.0 × 10^1^ GC/μL to 1.0 × 10^5^ GC/μL) of the corresponding synthetic DNA standard. All qPCR and RT-qPCR reactions were performed in duplicate on a Bio-Rad CFX Opus 96 Real-Time PCR System (Bio-Rad Laboratories, Inc., Singapore) using manual settings for threshold and baseline.

#### 2.4.3 Quality assurance controls

Quality assurance controls were included to assess viral recovery and PCR inhibition, according to published protocol by La Rosa et al. (33). Briefly, the concentration and extraction efficiency of the method was assessed through the viral recovery of vMC0 added to the samples prior to concentration phase (Sect. 2.4.1) and on the standard curve generated by serial 10-fold dilutions of vMC0 stock solution. Such efficiency (%) was calculated as *10^(ΔCq/m) x F x 100* where: ΔCq is the difference between the PCR quantification cycle (Cq) value of the reaction of each sample spiked with vMC0 and the Cq value of the reaction containing the extracted RNA of the vMC0; m is the slope of vMC0 standard curve; F is the fraction of the initial sample processed (equal to 1.125 in this study, as vMC0 was added to 45 mL of sample, of which 40 mL was recovered after the initial centrifugation step). The presence of PCR inhibition in each sample extract was assessed for both qPCR and RT-qPCR, using a HAdV qPCR assay as reference for DNA extracts and a SARS-CoV-2 RT-qPCR assay for the RNA extracts, respectively. Briefly, to test potential qPCR inhibition, two additional reactions were prepared by adding 1 μL of a synthetic HAdV control DNA (10^3^ GC/μL TaqMan Comprehensive Microbiota Control plasmid, Applied Biosystems) to sample extract and to molecular-grade water, that represent a non-inhibited reference reaction. An analogous procedure was applied to test potential RT-qPCR inhibition but using 1 μL of synthetic SARS-CoV-2 control RNA (10^3^ GC/μL kindly provided by the Italian National Institute of Health). Then, the difference between the Cq values from the two reactions was calculated according to the formula *Cq (sample + control DNA or RNA) – Cq (water + control DNA or RNA)*. The samples were considered inhibited when the result was > 2 (corresponding to an inhibition of the PCR greater than 75%) and the PCR assay was repeated using dilutions of the sample extract (e.g., 1:2, 1:5, 1:10).

#### 2.4.4 Norovirus sequencing

To investigate the NoVggII circulation on cruise ships, sequencing was performed on untreated samples collected from ships belonging to companies A and C (*n* = 7). These vessels were selected because the same ship from each company docked multiple times during the study period, so to understand the possible viral persistence onboard over time. cDNA samples were subjected to Sanger sequencing by Eurofins Genomics – GATC Biotech (Constance, Germany). The resulting nucleotide sequences were manually trimmed and exported in FASTA format, then analyzed using the Basic Local Alignment Search Tool (BLASTn, National Institutes of Health, United States) to identify the predominant NoVggII strain present in each wastewater sample on the basis of those recorded on GenBank database (National Center for Biotechnology Information, NCBI).^1^

### 2.5 Ship information and clinical data collection and analysis

Ships information was retrieved from Maritime Declaration of Health (MDH) as follows: (i) vessel details (e.g., name, flag, IMO number); (ii) port of arrival and destination; (iii) list of ports visited in the previous 30 days; (iv) number of passengers, crew members, and other people who had embarked.

#### 2.5.1 Clinical data categorization

Clinical data were obtained from medical records, covering the 7–10 days preceding arrival at the port. Medical records contained aggregated diagnostic codes for diseases, injuries, and causes of death, as classified according to either the International Classification of Diseases (ICD-10, WHO version) or, in some instances, the United States clinical modification (ICD-10-CM). To harmonize the analysis, ICD-10 (WHO)^2^ was used as the reference classification system. Medical codes associated with respiratory or gastrointestinal symptoms of likely viral infectious origin were selected according to the following three criteria: (i) conditions in which a viral etiology is explicitly indicated (e.g., J12.1 viral pneumonia due to respiratory syncytial virus; A08.11 acute gastroenteropathy due to norovirus); (ii) acute manifestations even when the etiological agent is unspecified (e.g., J22 unspecified acute lower respiratory infection; A09.0 infectious gastroenteritis and colitis, unspecified); and (iii) purely symptomatic conditions (R-codes), representing broader syndromic groupings that may be associated with viral infections (e.g., R05.1 acute cough; R19.7 diarrhea, unspecified). The categorization of the ICD-10 codes is reported in Supplementary Tables S2, S3 for respiratory and gastrointestinal symptoms, respectively. Whenever a code present in ICD-10-CM but not in ICD-10 WHO was encountered in the medical records, the specific code was included in the analysis if it met the above-mentioned criteria. It should be noted that medical codes are primarily assigned on the basis of syndromic diagnoses; however, some of the selected codes for respiratory symptoms refer explicitly to the identification of certain viral agents confirmed by diagnostic testing, namely seasonal influenza virus (J10 code), and COVID-19 (U07.1).

### 2.6 Data analysis

Microbiological data were log_10_-transformed to assess the performance of the wastewater treatment on board, that has been expressed as logarithmic reduction (LR). The LR was calculated using the formula *LR = Log_10_(N_in_/N_out_)*, where: N_in_ is the microbial concentrations of sewages before treatment (untreated sample) and N_out_ is the concentration measured after the treatment (treated sample), according to established approach for evaluating waterborne microorganism reductions using common water treatment technologies (34). For fecal indicator parameters, when the concentration was not detectable in sample after treatment, the abatement was calculated by giving a value equal to half of the detection limit, in accordance with a widely accepted practice in microbiological data analysis (35, 36). Graphical representation was generated using Excel for Windows (Microsoft Office Excel 2016, Redmond, Washington, United States).

## 3 Results

### 3.1 Viral pathogens analysis

The concentration and extraction procedure had an average recovery of 35.9% (ranging from a minimum of 2.1% to a maximum of 97.5%) as evaluated by spiking the wastewater samples with vMC0. qPCR inhibition, assessed by comparing Cq values of HAdV-spiked sample extracts and molecular-grade water, showed an average ΔCq of 0.32 (range: 0.00–1.33), with no samples exhibiting inhibition. Similarly, RT-qPCR using synthetic SARS-CoV-2 resulted in an average ΔCq of 0.66 (range: 0.00–3.75). Mild inhibition was observed in two untreated samples, with ΔCq values of 2.52 (Company A, 09/09/2024) and 3.75 (Company B, 02/10/2024). In both cases, inhibition was resolved following a 1:2 dilution of the sample extract.

**Figure 1:**
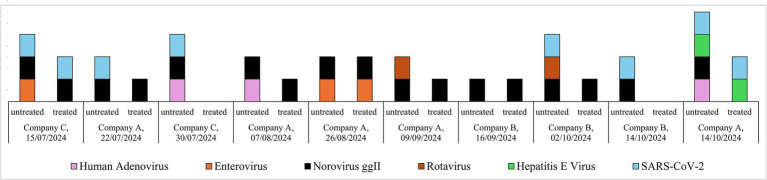
Occurrence of viral pathogens in treated and untreated samples of the 10 monitored ships (hepatitis A virus, influenza A virus and respiratory syncytial viruses were searched but not detected).

Untreated sewages showed a predominance of NoVggII (10/10, 100%), followed by SARS-CoV-2 (60%), HAdV (30%), enterovirus and rotavirus (20% each), and HEV (10%), whereas neither HAV, IAV, nor RSV genotypes were detected in any of the samples. After treatment, the occurrence of the detected viruses was reduced to 70% (7 out of 10 treated samples) for NoVggII, 30% for SARS-CoV-2, 10% for enterovirus and HEV (each), while none of the treated samples were positive for HAdV and rotavirus (Figure 1).

Quantitative results of pathogenic viruses during the entire monitoring period are summarized in Supplementary Table S4. The highest viral concentrations were observed for NoVggII in untreated samples, ranging from 2.9 × 10^3^ GC/L to 4.3 × 10^6^ GC/L (average 5.9 × 10^5^ GC/L). Similarly high concentrations were found for SARS-CoV-2, the second most frequently detected virus, with values ranging from 6.9 × 10^3^ GC/L to 7.9 × 10^5^ GC/L (average 1.6 × 10^5^ GC/L, calculated on positive samples). Interestingly, maximum levels of both viruses were detected on Company A vessels. Viral concentration of enterovirus and HEV in untreated samples were in the order of magnitude of 10^4^–10^5^ GC/L, whereas lower levels were observed for HAdV and rotavirus, except for a HAdV peak value of approximately 10^6^ GC/L detected on a Company A ship in October. Overall, viral concentrations in treated samples were reduced by less than 1 Log_10_ compared to their corresponding untreated samples, except for one ship for NoVggII (Company A, 22/07/24) and HEV (Company A, 14/10/24), where the viral load in the treated sample slightly exceeded that of the untreated one. Such a result is likely due to the inherent variability of environmental data in terms of sampling and analysis (37).

#### 3.1.1 Norovirus molecular characterization

NoVggII nucleotide sequencing was successfully obtained from the seven untreated samples from company A and C. Sequence alignment against the NCBI nucleotide database revealed percent identities ranging from 86 to 100% with previously deposited NoVggII strains, as summarized in Supplementary Table S5. Interestingly, in three consecutive dockings of a Company A ship, norovirus genotype GII.4 was identified and the sequenced strains showed high nucleotide similarity to GII.4 strains previously reported in South Africa.

### 3.2 Wastewater treatment system performance based on fecal indicators

**Figure 2:**
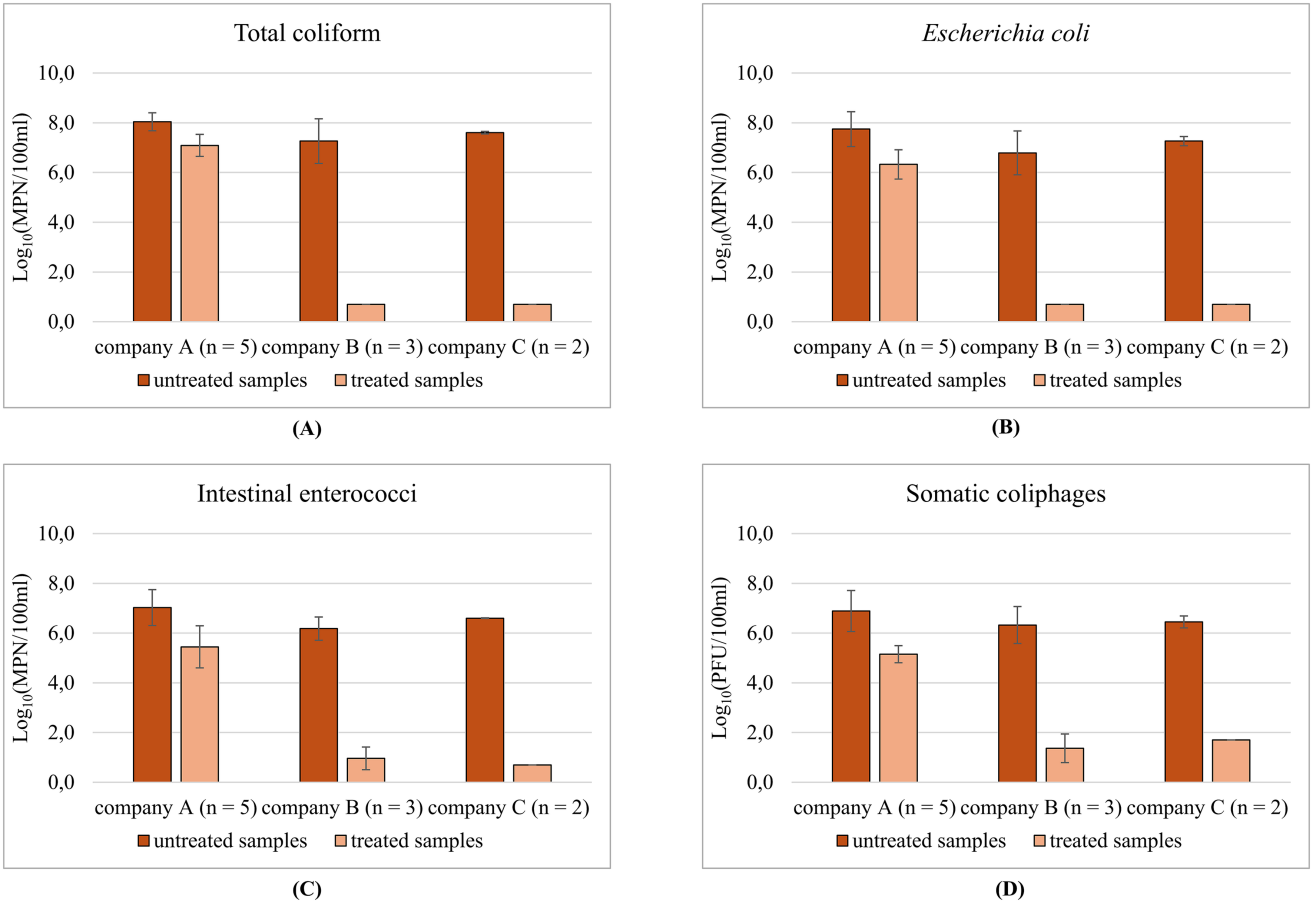
Microbial concentration in untreated and treated samples, separately for each shipping company: **(A)** total coliform, **(B)** *Escherichia coli*, **(C)** intestinal enterococci, and **(D)** somatic coliphages.

The fecal contamination in the untreated samples compares to typical fecal coliform concentrations at the entrance of domestic wastewater treatment plants, ranging between 10^6^ and 10^8^ MPN/100 mL (38). The comparison of untreated and treated samples revealed differences among shipping companies in terms of efficiency of wastewater treatments based on bacterial and viral indicators (Figure 2). The average microbial abatements obtained during the study period are reported in Table 1, separately for each microbial parameter. Overall, ships from Company A showed a mean reduction in microbial indicators less than 2 Log_10_, whereas most of the treated samples from companies B and C showed indicator concentrations below the detection limit, with abatements between 5 and 7 Log_10_.

**Table 1:** Bacterial and viral indicator removal by microbial parameter and shipping company (results are reported as Log_10_ reduction).

Microbial parameter	Shipping company	n. obs	Average microbial abatement	Microbial abatement range (min–max)
Total coliform	Company A	5	0.95	0.28–1.73
	Company B	3	6.56	5.54–6.91
	Company C	2	6.90	6.94 and 6.86
*Escherichia coli*	Company A	5	1.42	0.25–2.72
	Company B	3	6.09	5.10–6.79
	Company C	2	6.56	6.43 and 6.70
Intestinal enterococci	Company A	5	1.58	0.78–3.40
	Company B	3	5.22	4.68–5.95
	Company C	2	5.90	5.91 and 5.89
Somatic coliphages	Company A	5	1.74	0.94–2.41
	Company B	3	4.96	4.29–5.48
	Company C	2	4.75	4.58 and 4.92

### 3.3 Ship information and clinical data

Ship information, including number of persons onboard and aggregated clinical data, separately for respiratory and gastrointestinal symptoms, are presented in Table 2. Companies differ markedly in terms of onboard capacity: Companies A and C operate high-capacity vessels, with typical numbers between 3,000 and 5,000 persons, whereas Company B managed smaller ships, accommodating 500 to 1,000 persons (Cruise Lines International Association).^3^ All companies had called at Mediterranean ports within the 30 days preceding arrival at the monitoring port.

**Table 2:** Ship information and clinical surveillance.

Ship company, monitoring date	Total presences (passengers, crew)	Reference period of clinical surveillance	Countries visited in the last 30 days (alphabetic order)	Number of people with respiratory symptoms (possible viral origin)	Number of people with gastrointestinal symptoms (possible viral origin)
Company C, 15/07/2024	2,877 (2,105, 772)	03/07/24–14/07/24	France, Greece, Italy, Malta, Tunisia	12 (U07.2 for all cases)	7
Company A, 22/07/2024	2,870 (2079, 791)	12/07/24–22/07/24	France, Italy, Spain	27 (J10 for 10 cases and 1/10 identified as influenza B virus)	12 (only 3 cases tested for norovirus, all negative)
Company C, 30/07/2024	3,007 (2,228, 779)	14/07/24–28/07/24	Croatia, Greece, Italy, Montenegro	23 (U07.2 for all cases)	0
Company A, 07/08/2024	5,731 (3,955, 1776)	30/07/24–06/08/24	France, Italy, Spain	5	2
Company A, 26/08/2024	2,837 (2047, 790)	14/08/24–24/08/24	France, Italy, Spain	7	6
Company A, 09/09/2024	4,937 (3,743, 1,194)	29/08/24–08/09/24	France, Italy, Spain	10 (U07.1 for 1 case)	6
Company B, 16/09/2024	665 (374, 291)	06/09/24–14/09/24	France, Italy	0	0
Company B, 02/10/2024	964 (545, 419)	20/09/24–30/09/24	France, Italy, Monaco, Spain	15 (U07.1 for 3 cases)	3
Company B, 14/10/2024	954 (557, 397)	04/10/24–11/10/24	France, Italy, Monaco, Portugal, Spain	13 (U07.1 for 1 case)	4
Company A, 14/10/2024	4,924 (3,743, 1,181)	06/10/24–12/10/24	France, Italy, Spain	8 (U07.1 for 1 case)	1

Number of clinical cases are reported according to standardized medical codes selected for presumptive infectious origin. Codes assigned to identified respiratory pathogens are explicitly indicated. J10, influenza due to identified seasonal influenza virus; U07.1, COVID-19 virus identified; U07.2 COVID-19, virus not identified.

**Figure 3:**
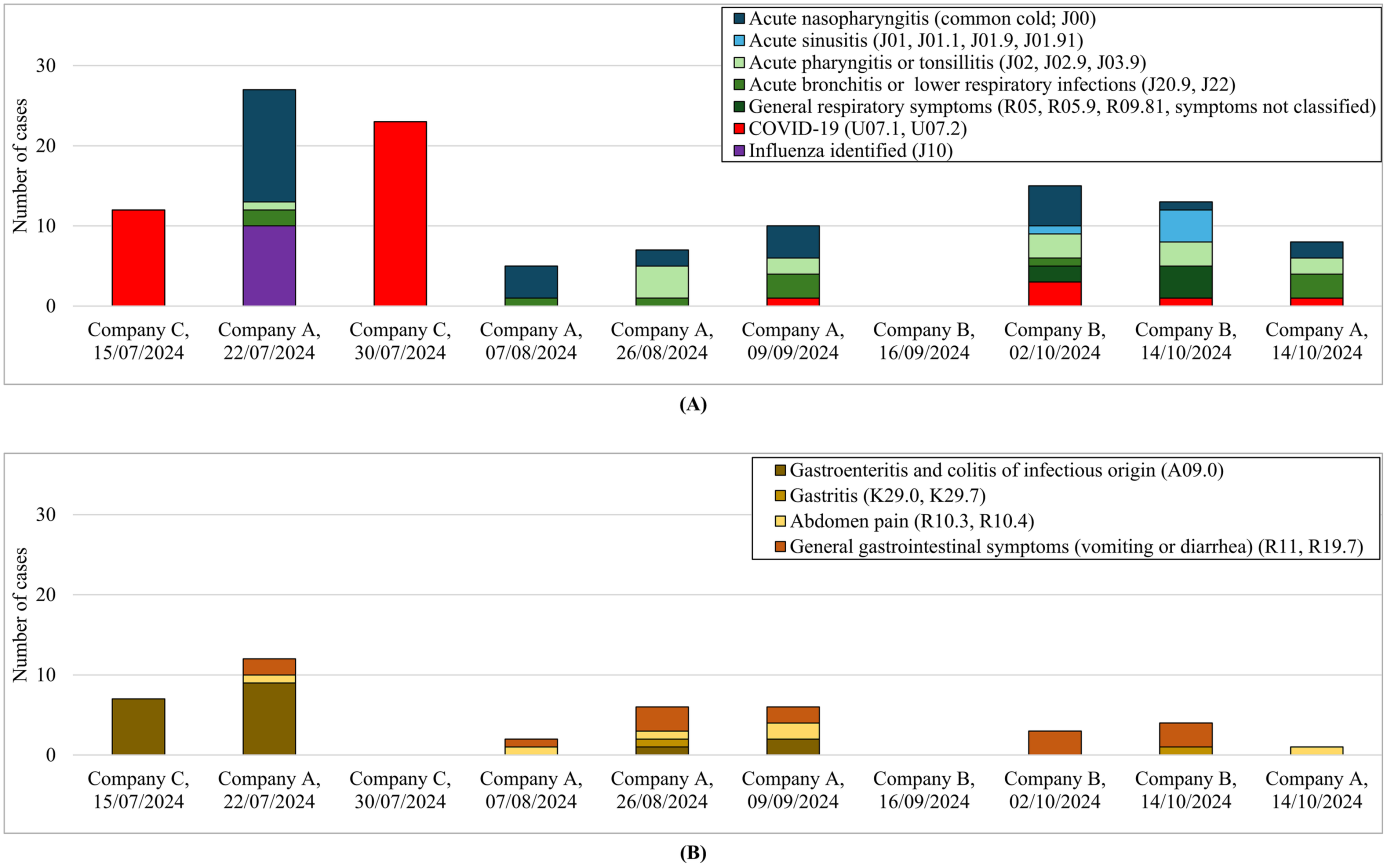
Categorization of syndromes of potential viral infectious origin identified during the surveillance period: **(A)** respiratory symptoms and **(B)** gastrointestinal symptoms.

The complete set of medical codes identified during the surveillance period (7–10 days preceding arrival at the port) for respiratory and gastrointestinal syndromes of potential viral infectious origin, together with the distribution of cases by ship and sampling date, is represented in Figure 3 and detailed in Supplementary Tables S6, S7, respectively. For respiratory syndromes, the identified codes included common cold (J00), acute sinusitis (J01, including subcodes J01.0 and J01.9), acute pharyngitis (J02.9), acute tonsillitis (J03.9), acute bronchitis (J20.9), acute lower respiratory infection (J22), and cough (R05, R05.9), as well as influenza (J10) and COVID-19 (U07.1 and U07.2). For gastrointestinal syndromes, the relevant codes comprised infectious gastroenteritis and colitis (A09.0), acute gastritis (K29.0), unspecified gastritis (K29.7), abdominal pain (R10.3 and R10.4), nausea and vomiting (R11), and unspecified diarrhea (R19.7). Overall, clinical surveillance identified more respiratory than gastrointestinal cases. Etiological identification through diagnostic testing was available for a limited number of pathogens (SARS-CoV-2, influenza virus, and norovirus) and for 5 out of 10 ships: four ships reported COVID-19 cases coded as U07.1, and one ship reported influenza cases (J10), further identified as influenza B virus. Norovirus testing was performed on one ship in selected cases but yielded negative results (Table 2).

### 3.4 Qualitative evaluation of wastewater and clinical data

Agreement between wastewater and clinical data was evaluated at the ship-arrival level for SARS-CoV-2, influenza virus, and norovirus, for which virological confirmation was available, although only for a limited number of cases (Section 3.3, Table 2).

For SARS-CoV-2, agreement between untreated wastewater detection and COVID-19 cases with identified virus (U07.1) was observed in 75% (3/4) of ship arrivals (Company B, 02/10/24 and 14/10/24; Company A, 14/10/24), as detailed in Table 3. SARS-CoV-2 was also detected in wastewater samples from two vessels hosting passengers with clinically diagnosed COVID-19 cases without virological identification (U07.2) (Company C, 15/07/24 and 30/07/24), supporting the presence of viral circulation despite the absence of confirmed diagnosis. Thus, the proportion of ship arrivals showing agreement between wastewater and clinical data increased to 83% (5/6) when both confirmed and suspected clinical diagnoses were considered.

**Table 3:** Qualitative comparison between SARS-CoV-2 detection in untreated wastewater and clinical COVID-19 cases at each ship arrival during the study period.

Ship arrival	SARS-CoV-2 in wastewater (yes/no)	COVID-19 cases (suspected, confirmed, not reported)	Interpretation of wastewater and clinical data
Company C, 15/07/2024	Yes	Suspected	Consistent with suspected clinical circulation
Company A, 22/07/2024	Yes	Not reported	Wastewater detection without reported clinical cases
Company C, 30/07/2024	Yes	Suspected	Consistent with suspected clinical circulation
Company A, 07/08/2024	No	Not reported	Neither wastewater nor clinical evidence
Company A, 26/08/2024	No	Not reported	Neither wastewater nor clinical evidence
Company A, 09/09/2024	No	Confirmed	No wastewater detection despite reported clinical cases
Company B, 16/09/2024	No	Not reported	Neither wastewater nor clinical evidence
Company B, 02/10/2024	Yes	Confirmed	Wastewater detection with confirmed clinical cases
Company B, 14/10/2024	Yes	Confirmed	Wastewater detection with confirmed clinical cases
Company A, 14/10/2024	Yes	Confirmed	Wastewater detection with confirmed clinical cases

Confirmed, COVID-19 with virus identification through diagnostic testing (U07.1); Suspected, clinically diagnosed COVID-19 without viral confirmation (U07.2).

For influenza virus, IAV was not detected in untreated samples. However, one ship (Company A, 22/07/24) reported 10 influenza-confirmed cases (J10), including one case with the identification of influenza B virus. This discrepancy may reflect the specific influenza strain involved, namely IBV rather than IAV, which was the target of wastewater analysis.

Regarding enteric viruses, norovirus was frequently detected in untreated wastewater samples. However, systematic diagnostic testing for norovirus was not consistently performed among symptomatic individuals. In particular, on one vessel (Company A, 22/07), diagnostic testing for norovirus was limited to 3 of the 12 individuals presenting gastrointestinal symptoms, all of whom tested negative, despite norovirus was detected in the corresponding wastewater sample. Consequently, the limited availability of virological confirmation among symptomatic cases may have led to underestimation of agreement between wastewater and clinical data for enteric pathogens.

## 4 Discussion

### 4.1 Wastewater and clinical surveillance

Our study revealed the consistent detection of norovirus and SARS-CoV-2 in untreated sewage samples, along with the occurrence of other pathogenic viruses, that is, HAdV, enterovirus, HEV, and rotavirus. The little body of knowledge on wastewater surveillance from cruise ships makes comparisons with existing scientific literature limited. Nevertheless, our results partially align with those reported by Jones et al. (18), who investigated passenger ships operating between the United Kingdom and Ireland in 2022. In their study, only SARS-CoV-2 and norovirus were detected, that were also the most frequently identified viruses in our samples. However, their overall detection rate was below 10%, likely due to differences in vessel type (commercial rather than cruise ships). Moreover, the authors attributed the low frequency of norovirus to the residual impact of non-pharmaceutical interventions implemented during the COVID-19 pandemic, suggesting that higher detection rates might be observed in the pre- or post-pandemic period (18). In fact, during gastrointestinal outbreaks on international cruise ships in Netherlands, environmental swabs revealed that all tested bathroom surfaces were positive for norovirus (39).

In our study, most of the norovirus strains detected in sewages belong to GII.4 genotype, which is the predominant human-associated strain circulating globally (40). Moreover, norovirus-positive sewage samples collected from the same vessel during five separate dockings revealed that several GII.4 strains shared high sequence similarity with isolates previously reported in South Africa, despite the fact that all cruise itineraries in this study were confined to European ports (Italy, France, and United Kingdom). This finding can be explained from an epidemiological and environmental perspective. Cruise ship crews often include personnel from diverse geographical origins; thus, infected individuals may introduce viral strains that persist and circulate onboard across multiple voyages. Although we did not identify previous reports of European outbreaks directly sustained by South African strains, GII.4 variants are well known for their capacity to emerge in distant regions and rapidly spread worldwide, with novel lineages repeatedly introduced and documented also in Europe (40, 41). In fact, norovirus is highly stable in the environment and can persist on surfaces and indoor settings for long time (42, 43). As an example, in the United States, Hall et al. (44) reported that recurrent outbreaks on vessels were attributable to the recrudescence of viral contamination from one sailing to the next.

The integration of wastewater monitoring with onboard clinical surveillance (although partial) provided pathogen-specific insights. A 75% agreement was observed between SARS-CoV-2 detection in untreated wastewaters and COVID-19 cases with viral identification. This proportion increased up to 83% (5/6 ship arrivals) when vessels reporting clusters of clinically diagnosed COVID-19 cases without viral identification were also considered. These findings support the potential role of wastewater surveillance as a complementary monitoring tool in maritime settings. In contrast, agreement was not observed for influenza virus, but the interpretation may have been influenced by strain-specific detection (influenza B vs. influenza A). On the other hand, agreement between wastewater and clinical data could not be assessed for enteric viruses due to the incomplete or lack of clinical diagnostic testing among individuals with gastrointestinal symptoms, despite their frequent detection in untreated wastewater, with norovirus identified in 100% of the samples and HAdV, enterovirus, rotavirus, and HEV in 10–30% of samples.

### 4.2 Microbiological performance of onboard wastewater treatment systems

A critical observation from this study is the wide variation in microbial reduction performance among ships, likely linked to differences in onboard sanitation systems. For example, companies B and C vessels demonstrated complete or near-complete removal of microbial parameters in treated effluents, achieving reductions of ≥5 Log_10_. In contrast, Company A ships showed considerably lower treatment efficacy, with detectable viral loads and high microbial concentrations even in treated wastewaters. Compliance with sewage discharge is regulated under MARPOL Annex IV, which permits operational discharge for ships equipped with an approved sewage treatment plant, provided that the effluent does not produce visible floating solids or discoloration of the surrounding water. The approval of such systems is based on the performance criteria established in the IMO Revised guidelines on implementation of effluent standards and performance tests for sewage treatment plants (45). These criteria are assessed during type-approval testing under controlled conditions, based on a minimum number of effluent samples and expressed as geometric means for thermotolerant coliforms, total suspended solids (TSS), biochemical oxygen demand (BOD5), chemical oxygen demand (COD), and pH. In the present study, only microbiological indicators were assessed under real operating conditions. The geometric mean concentration of *E. coli* (used here as a representative thermotolerant coliform indicator) in treated effluent from Company A was approximately 10^6^ MPN/100 mL, exceeding the 100 MPN/100 mL benchmark established for certification testing. However, because the complete set of performance parameters was not evaluated, these findings should be interpreted as indicating reduced microbiological removal efficiency rather than definitive regulatory non-compliance. These results highlight a substantial heterogeneity in the performance of sewage treatments on different ships, whose public health implications need to be further investigated.

### 4.3 Considerations for wastewater surveillance in maritime outbreak management

Public health management in maritime settings relies on International Health Regulations (IHR) that identify core capacity related to surveillance and response to infectious disease at designated points of entry (46). In this context, wastewater monitoring on cruise ships could be incorporated into the event management workflow, which encompasses event detection by the competent port authorities at port and is followed by a sequence of structured actions (i.e., verification, risk assessment, adoption of public health measures for the infectious diseases, and communication) requiring coordinated efforts among ship operators, port authorities, and subsequent ports of call (47). The surveillance of wastewater may serve as an additional source of information at the stage of event detection and can also contribute to the evaluation of the effectiveness of response measures in reducing pathogen circulation. Cruise operators could designate trained onboard personnel to collect standardized wastewater samples at docking (or during the pre-arrival reporting window), while port health authorities, supported by designed laboratories, would coordinate analysis and reporting within defined timeframes. Wastewater results could then be incorporated into the existing outbreak management and notification procedures for passenger ships and aligned with established pre-arrival reporting models (2, 3, 47). If wastewater signals indicate increased viral circulation (e.g., repeated detection or marked increases in specific viral targets), realistic and proportionate responses could include: (i) enhanced onboard case finding and diagnostic testing; (ii) reinforcement of hygiene, isolation, and environmental disinfection measures, as specified in shipboard outbreak management protocols; (iii) structured communication with the next port of call; and (iv) targeted technical verification of wastewater treatment system performance.

### 4.4 Limitations

Given the pilot nature of the study, the investigation was conducted at a single seaport and over a limited time frame, which may restrict the generalizability of the results to other maritime contexts. Then, the comparison between viral detection in wastewater and clinical cases remained qualitative, as onboard clinical surveillance is largely syndromic and diagnostic ascertainment is available for only a limited number of pathogens, and even for these pathogens, diagnostic testing was incomplete (e.g., SARS-CoV-2). This limitation may have led to underestimation of agreement between environmental and clinical data. Finally, specific technical details on the design of each ships’ wastewater treatment system were not available, precluding a full assessment of their treatment performance. In fact, heterogeneity in system configuration, hydraulic treatment capacity relative to actual loading conditions, and disinfectant management may have contributed to the variability observed among vessels, but such information was not accessible for the present study.

## 5 Conclusion

This pilot study supports a two-level set of conclusions, both of which are relevant for strengthening preparedness against future pandemics:

- *Integration of surveillance approaches*. Wastewater surveillance, when combined with onboard clinical surveillance, is able to detect the circulation of enteric and respiratory pathogens, including SARS-CoV-2, norovirus, human adenovirus, enterovirus, hepatitis E virus, and rotavirus. Clinical surveillance alone is not sufficient to promptly identify such circulation, especially when diagnostic testing is incomplete or inconsistently applied. This finding supports the use of wastewater monitoring to inform evidence-based strategies for outbreak prevention in the context of international maritime traffic, in line with recent initiatives promoted by the European Health Emergency Preparedness and Response Authority (HERA).
- *Performance of sewage treatment systems*. The analysis of treated sewage revealed marked variability in wastewater treatment efficacy across vessels, with potential implications for both human health and the environment. These findings underscore the need for greater transparency and harmonization of technical specifications and performance for maritime wastewater treatment. Such efforts would also be in accordance with existing international frameworks, such as the IHR on management of public health risks by port health authorities and MARPOL Convention on ship pollution.

## Data Availability

The original contributions presented in the study are included in the article/Supplementary material, further inquiries can be directed to the corresponding author.

## Footnotes

1
https://blast.ncbi.nlm.nih.gov/Blast.cgi

2
https://icd.who.int/browse10/2019/en

3
https://cruising.org/
