# Supplementary material for: “Navigating Healthy Waters”: monitoring ship wastewater as a key defense against infectious diseases—a pilot study on a Mediterranean seaport
Source: Front Public Health. 2026 Apr 23;14:1786232. doi: 10.3389/fpubh.2026.1786232 (PMC13149468; doi:10.3389/fpubh.2026.1786232)
Supplement: Supplementary file 1 [file Supplementary_file_1.docx]

Supplementary Material

# Supplementary tables: list of content

Table S1. Oligonucleotide primers and probes used for viral detection by real time (RT)-qPCR, thermal cycling protocol, and references. Target regions are also reported for each viral parameter (if available).

Table S2. Categorization of medical codes from International Classification Disease (ICD) for respiratory symptoms of potential viral infectious origin. The codes have been divided according to the ICD-10 chapter (WHO, Version 2019)

Table S3. Categorization of medical codes from International Classification Disease (ICD) for gastrointestinal symptoms of potential viral infectious origin. The ICD-10 categories have been divided according to the ICD-10 chapters (WHO, Version 2019)

Table S4. Viral concentration of the detected target during the study period (hepatitis A virus, influenza A virus, respiratory syncytial virus A and B were searched but not detected)

Table S5. Identification of NoVggII strain detected in Companies A and C

Table S6. Medical codes for respiratory syndromes of potential viral infectious origin recorded during the study period. Numbers indicate the cases reported in medical consultation report for the 7–10 days preceding arrival at the port

Table S7. Medical codes for gastrointestinal syndromes of potential viral infectious origin recorded during the study period. Numbers indicate the cases reported in medical consultation report for the 7–10 days preceding arrival at the port

Table S1. Oligonucleotide primers and probes used for viral detection by real time (RT)-qPCR, thermal cycling protocol, and references. Target regions are also reported for each viral parameter (if available).

| Primer or probe names | Concentration (µM) | Sequence (5’-3’) | Thermal cycle | Original Reference |
| --- | --- | --- | --- | --- |
| **Human adenovirus (hexon gene)** | | | | |
| Primer AdF | 0.9 | CWT ACA TGC ACA TCK CSG G | 50° C: 2 min, 95° C: 10 min, 45 cycles (95° C: 15 s; 60° C: 1 min) | Hernroth et al. 2002 |
| Primer AdR | 0.9 | CRC GGG CRA AYT GCA CCA G |  |  |
| Probe AdP | 0.225 | FAM–CCG GGC TCA GGT ACT CCG AGG CGT CCT–TAMRA |  |  |
| **Enterovirus (5′ UTR region)** | | | | |
| Primer EVF | 0.6 | GGC CCC TGA ATG CG GCT AAT | 48° C: 30 min, 95° C: 10 min, 45 cycles (95° C: 15 s; 60° C: 1 min) | Donaldson et al. 2002 |
| Primer EVR | 0.6 | CAC CGG ATG GCC AAT CCA A |  |  |
| Probe EV | 0.25 | FAM–CGG ACA CCC AAA GTA GTC GGT TCC G–TAMRA |  |  |
| **SARS-CoV-2 (ORF1ab)** | | | | |
| 2297-CoV-2F | 0.5 | ACA TGG CTT TGA GTT GAC ATC T | 50° C: 30 min, 95° C: 5 min, 45 cycles (95° C: 15 s; 60° C: 30s) | La Rosa et al. 2021 |
| 2298-CoV-2R | 0.9 | AGC AGT GGA AAA GCA TGT GG |  |  |
| 2299-CoV-2P | 0.25 | FAM–CAT AGA CAA CAG GTG CGC TC–MGBEQ |  |  |
| **Norovirus genogroup II (ORF2 region)** | | | | |
| QNIF2 | 0.5 | ATG TTC AGR TGG ATG AGR TTC TCW GA | 55° C: 60 min, 95° C: 5 min, 45 cycles (95° C: 15 s; 60° C: 1 min; 65°C: 1 min) | ISO 15216-1:2017 |
| COG2R | 0.9 | TCG ACG CCA TCT TCA TTC ACA |  |  |
| QNIFS | 0.25 | FAM–AGC ACG TGG GAG GGC GAT CG–TAMRA |  |  |
| **Hepatitis A virus (5′ UTR region)** | | | | |
| HAV68 | 0.5 | TCA CCG CCG TTT GCC TAG | 55° C: 60 min, 95° C: 5 min, 45 cycles (95° C: 15 s; 60° C: 1 min; 65°C: 1 min) | ISO 15216-1:2017 |
| HAV24 | 0.9 | GGA GAG CCC TGG AAG AAA G |  |  |
| HAV150(-) | 0.25 | FAM–CCT GAA CCT GCA GGA ATT AA–MGB |  |  |
| **Hepatitis E virus (ORF2/3)** | | | | |
| JVHEVF | 0.5 | GGT GGT TTC TGG GGT GAC | 50° C: 60 min, 95° C: 5 min, 45 cycles (95° C: 15 s; 60° C: 1 min; 65°C: 1 min) | Iaconelli et al. 2020 |
| JVHEVR | 0.9 | AGG GGT TGG TTG GAT GAA |  |  |
| JVHEVPmod | 0.25 | FAM – TGA TTC TCA GCC CTT CGC – MGB |  |  |
| **Rotavirus (NSP3)** | | | | |
| NVP3-R1 | 0.5 | GGT CAC ATA ACG CCC CTA TA | 50° C: 30 min, 95° C: 10 min, 45 cycles (95° C: 15 s; 60° C: 45 s) | Freeman et al. 2008 |
| NVP3-FDeg | 0.9 | ACC ATC TWC ACR TRA CCC TC |  |  |
| NVP3-Probe | 0.25 | FAM – ATG AGC ACA ATG TTA AAA GCT AAC ACT GTC AA – MGB |  |  |
| **Influenza A virus** | | | | |
| TaqMan® Microbial Assays | assay ID Vi99990011_po (custom TaqMan Assays; Thermo Fisher Scientific, USA) * | | 25°C: 2 min, 50°C: 15 min, 95°C: 2 min, 45 cycles (95°C: 3 s, 60°C: 1 min) | Ahmed et al. 2023 |
| **Respiratory syncytial virus A** | | | | |
| TaqMan® Microbial Assays | assay ID Vi99990014_po (custom TaqMan Assays; Thermo Fisher Scientific, USA) * | | 25°C: 2 min, 50°C: 15 min, 95°C: 2 min, 45 cycles (95°C: 3 s, 60°C: 1 min) | Ahmed et al. 2023 |
| **Respiratory syncytial virus B** | | | | |
| TaqMan® Microbial Assays | assay ID Vi99990015_po (custom TaqMan Assays; Thermo Fisher Scientific, USA) * | | 25°C: 2 min, 50°C: 15 min, 95°C: 2 min, 45 cycles (95°C: 3 s, 60°C: 1 min) | Ahmed et al. 2023 |

* The composition of the reaction mix, including primer and probe sequences, is covered by a Thermo Fisher patent

References for the Table S1

Ahmed W et al. Occurrence of multiple respiratory viruses in wastewater in Queensland, Australia: potential for community disease surveillance. Sci Total Environ. 2023;864:161023. doi:10.1016/j.scitotenv.2022.161023.

Donaldson KA et al. Detection, quantitation and identification of enteroviruses from surface waters and sponge tissue from the Florida Keys using real-time RT-PCR. Water Res. 2002;36(10):2505–2514. doi:10.1016/S0043-1354(01)00479-1.

Freeman MM et al. Enhancement of detection and quantification of rotavirus in stool using a modified real-time RT-PCR assay. J Med Virol. 2008;80(8):1489–1496.

Hernroth BE et al. Environmental factors influencing human viral pathogens and their potential indicator organisms in the blue mussel, Mytilus edulis: the first Scandinavian report. Appl Environ Microbiol. 2002;68:4523–4533. doi:10.1128/AEM.68.9.4523-4533.2002.

Iaconelli M et al. Nine-year nationwide environmental surveillance of hepatitis E virus in urban wastewaters in Italy (2011–2019). Int J Environ Res Public Health. 2020;17:2059. doi:10.3390/ijerph17062059.

International Organization for Standardization. Microbiology of the food chain—Horizontal method for determination of hepatitis A virus and norovirus using real-time RT-PCR. Part 1: Method for quantification. ISO 15216-1:2017. Geneva: ISO; 2017.

La Rosa G et al. SARS-CoV-2 has been circulating in Northern Italy since December 2019: evidence from environmental monitoring. Sci Total Environ. 2021;750:141711.

Table S2. Categorization of medical codes from International Classification Disease (ICD) for respiratory symptoms of potential viral infectious origin. The codes have been divided according to the ICD-10 chapter (WHO, Version 2019)

| **ICD-10 Category** | **Codes and descriptor** | **Subcodes (if available)** |
| --- | --- | --- |
| **X. Diseases of the respiratory system (J00–J99)** | | |
| J00–J06 Acute upper respiratory infections | J00 Acute nasopharyngitis [common cold] | – |
|  | J01 Acute sinusitis | J01.0 Acute maxillary sinusitis; J01.1 Acute frontal sinusitis; J01.2 Acute ethmoidal sinusitis; J01.3 Acute sphenoidal sinusitis; J01.4 Acute pansinusitis; J01.9 Acute sinusitis, unspecified |
|  | J02 Acute pharyngitis | J02.8 Acute pharyngitis due to other specified organisms; J02.9 Acute pharyngitis, unspecified |
|  | J03 Acute tonsillitis | J03.8 Acute tonsillitis due to other specified organisms; J03.9 Acute tonsillitis, unspecified |
|  | J04 Acute laryngitis and tracheitis | J04.0 Acute laryngitis; J04.1 Acute tracheitis; J04.2 Acute laryngotracheitis; J04.3 Supraglottitis; J04.9 Acute laryngitis and tracheitis, unspecified |
|  | J05 Acute obstructive laryngitis (croup) and epiglottitis | J05.0 Acute obstructive laryngitis [croup]; J05.1 Acute epiglottitis; J05.9 Acute obstructive laryngitis, unspecified |
|  | J06 Acute upper respiratory infections of multiple and unspecified sites | J06.0 Acute laryngopharyngitis; J06.8 Other specified acute upper respiratory infections; J06.9 Acute upper respiratory infection, unspecified |
| J09-J18 Influenza and pneumonia | J09 Influenza due to identified zoonotic or pandemic influenza virus | – |
|  | J10 Influenza due to identified seasonal influenza virus | – |
|  | J11 Influenza, virus not identified | – |
|  | J12 Viral pneumonia, not elsewhere classified | J12.0 Adenoviral pneumonia; J12.1 Respiratory syncytial virus pneumonia; J12.2 Parainfluenza virus pneumonia; J12.3 Human metapneumovirus pneumonia; J12.8 Other viral pneumonia; J12.9 Viral pneumonia, unspecified |
|  | J17 Pneumonia in diseases classified elsewhere | J17.1 Pneumonia in viral diseases classified elsewhere |
|  | J18 Pneumonia, organism unspecified | J18.0 Bronchopneumonia, unspecified; J18.1 Lobar pneumonia, unspecified; J18.2 Hypostatic pneumonia, unspecified; J18.8 Other pneumonia, organism unspecified; J18.9 Pneumonia, unspecified |
| J20–J22 Acute lower respiratory infections | J20 Acute bronchitis | J20.3 Coxsackievirus bronchitis; J20.4 Parainfluenza virus bronchitis; J20.5 Respiratory syncytial virus bronchitis; J20.6 Rhinovirus bronchitis; J20.7 Echovirus bronchitis; J20.8 Acute bronchitis due to other specified organisms; J20.9 Acute bronchitis, unspecified |
|  | J21 Acute bronchiolitis | J21.0 Acute bronchiolitis due to respiratory syncytial virus; J21.1 Acute bronchiolitis due to human metapneumovirus; J21.8 Acute bronchiolitis due to other specified organisms; J21.9 Acute bronchiolitis, unspecified |
|  | J22 Unspecified acute lower respiratory infection | – |
| **XVIII. Symptoms, signs and abnormal clinical and laboratory findings (R00–R99)** | | |
| R00-R09 Symptoms and signs involving the circulatory and respiratory systems | R05 Cough | – |
| **XXII. Codes for special purposes (U00–U85)** | | |
| U00-U49 Provisional assignment of new diseases of uncertain etiology or emergency use | U04 Severe acute respiratory syndrome [SARS] | U04.9 Severe acute respiratory syndrome [SARS], unspecified |
|  | U07 Emergency use of U07 | U07.1 COVID-19, virus identified  U07.2 COVID-19, virus not identified |

Table S3. Categorization of medical codes from International Classification Disease (ICD) for gastrointestinal symptoms of potential viral infectious origin. The ICD-10 categories have been divided according to the ICD-10 chapters (WHO, Version 2019)

| **ICD-10 Category** | **Included codes and descriptor** | **Included subcodes (if available)** |
| --- | --- | --- |
| **I. Certain infectious and parasitic diseases (A00–B99)** | | |
| A00-A09 Intestinal infectious diseases | A08 Viral and other specified intestinal infections | A08.0 Rotaviral enteritis; A08.1 Acute gastroenteropathy due to Norwalk agent; A08.2 Adenoviral enteritis; A08.3 Other viral enteritis; A08.4 Viral intestinal infection, unspecified |
|  | A09 Other gastroenteritis and colitis of infectious and unspecified origin | A09.0 Other and unspecified gastroenteritis and colitis of infectious origin |
| **XI. Diseases of the digestive system (K00–K93)** | | |
| K20-K31 Diseases of oesophagus, stomach and duodenum | K29 Gastritis and duodenitis | K29.0 Acute haemorrhagic gastritis; K29.1 Other acute gastritis; K29.7 Gastritis, unspecified; K29.8 Duodenitis; K29.9 Gastroduodenitis, unspecified |
| **XVIII. Symptoms, signs and abnormal clinical and laboratory findings (R00–R99)** | | |
| R10-R19 Symptoms and signs involving the digestive system and abdomen | R10 Abdominal and pelvic pain | R10.3 Pain localized to other parts of lower abdomen; R10.4 Other and unspecified abdominal pain |
|  | R11 Nausea and vomiting | R11 Nausea and vomiting |
|  | R19 Other symptoms and signs involving the digestive system and abdomen | R19.7 Diarrhoea, unspecified |

Table S4. Viral concentration of the detected target during the study period (hepatitis A virus, influenza A virus, respiratory syncytial virus A and B were searched but not detected)

| **Company type** | **Sampling date** | **Sampling point** | **HAdV (GC/L)** | **EV (GC/L)** | **NoVggII (GC/L)** | **RV (GC/L)** | **HEV (GC/L)** | **SARS-CoV-2 (GC/L)** |
| --- | --- | --- | --- | --- | --- | --- | --- | --- |
| Company C | 15/07/24 | untreated | undet | 1.3 x 10^4^ | 4.6 x 10^3^ | undet | undet | 6.1 x 10^4^ |
|  |  | treated | undet | undet | undet | undet | undet | undet |
| Company A | 22/07/24 | untreated | undet | undet | 2.7 x 10^5^ | undet | undet | 1.5 x 10^4^ |
|  |  | treated | undet | undet | 6.3 x 10^5^ | undet | undet | undet |
| Company C | 30/07/24 | untreated | 1.1 x 10^3^ | undet | 1.2 x 10^4^ | undet | undet | 2.3 x 10^4^ |
|  |  | treated | undet | undet | undet | undet | undet | undet |
| Company A | 07/08/24 | untreated | 4.2 x 10^3^ | undet | 2.9 x 10^3^ | undet | undet | undet |
|  |  | treated | undet | undet | 9.4 x 10^2^ | undet | undet | undet |
| Company A | 26/08/24 | untreated | undet | 1.7 x 10^5^ | 2.4 x 10^4^ | undet | undet | undet |
|  |  | treated | undet | 1.1 x 10^4^ | undet | undet | undet | undet |
| Company A | 09/09/24 | untreated | undet | undet | 4.3 x 10^6^ | 2.6 x 10^3^ | undet | undet |
|  |  | treated | undet | undet | 1.5 x 10^6^ | undet | undet | undet |
| Company B | 16/09/24 | untreated | undet | undet | 4.6 x 10^3^ | undet | undet | undet |
|  |  | treated | undet | undet | undet | undet | undet | undet |
| Company B | 02/10/24 | untreated | undet | undet | 8.9 x 10^3^ | 9.4 x 10^2^ | undet | 6.9 x 10^3^ |
|  |  | treated | undet | undet | 1.8 x 10^3^ | undet | undet | undet |
| Company B | 14/10/24 | untreated | undet | undet | 6.1 x 10^5^ | undet | undet | 5.2 x 10^4^ |
|  |  | treated | undet | undet | undet | undet | undet | undet |
| Company A | 14/10/24 | untreated | 5.4 x 10^6^ | undet | 6.4 x 10^5^ | undet | 4.1 x 10^4^ | 7.9 x 10^5^ |
|  |  | treated | undet | undet | undet | undet | 1.8 x 10^5^ | 1.0 x 10^4^ |

EV = enterovirus; HAdV = Human Adenovirus; HEV = hepatitis E virus; NoVggII = norovirus genogroup II; RV = Rotavirus

Table S5. Identification of NoVggII strain detected in Companies A and C

| Company type | Sampling date | Accession number of the closest strain on NCBI data bank | Year and country of norovirus strain on NCBI data bank | Percentage of identity |
| --- | --- | --- | --- | --- |
| Company C | 15/07/24 | GII.4/MZ285612 | 2017, Brazil | 86% |
| Company A | 22/07/24 | GII.21/MH702285 | 2018, Thailand | 94% |
| Company C | 30/07/24 | GII.4/MF140664 | 2010, The Netherlands | 100% |
| Company A | 07/08/24 | GII.4/OP310003 | 2018, South Africa | 95% |
| Company A | 26/08/24 | GII.4/OP308356 | 2018, South Africa | 100% |
| Company A | 09/09/24 | GII.4/KJ710245 | 2011, South Africa | 90% |
| Company A | 14/10/24 | GII/MK733204 | 2017, Argentina | 100% |

Table S6. Medical codes for respiratory syndromes of potential viral infectious origin recorded during the study period. Numbers indicate the cases reported in medical consultation report for the 7–10 days preceding arrival at the port

| Medical codes identified in the surveillance period for respiratory symptoms | Company type and date of monitoring during 2024 | | | | | | | | | |
| --- | --- | --- | --- | --- | --- | --- | --- | --- | --- | --- |
|  | C, 15/07 | A, 22/07 | C, 30/07 | A, 07/08 | A, 26/08 | A, 09/09 | B, 16/09 | B, 02/10 | B, 14/10 | A, 14/10 |
| (J00) Acute nasopharyngitis [common cold] | 0 | 14 | 0 | 4 | 2 | 4 | 0 | 5 | 1 | 2 |
| (J01) Acute sinusitis | 0 | 0 | 0 | 0 | 0 | 0 | 0 | 0 | 2 | 0 |
| (J01.0) Acute maxillary sinusitis | 0 | 0 | 0 | 0 | 0 | 0 | 0 | 0 | 1 | 0 |
| (J01.9) Acute sinusitis, unspecified | 0 | 0 | 0 | 0 | 0 | 0 | 0 | 1 | 0 | 0 |
| (J01.91) Acute recurrent sinusitis, unspecified | 0 | 0 | 0 | 0 | 0 | 0 | 0 | 0 | 1 | 0 |
| (J02) Acute pharyngitis | 0 | 0 | 0 | 0 | 4 | 2 | 0 | 0 | 3 | 2 |
| (J02.9) Acute pharyngitis, unspecified | 0 | 0 | 0 | 0 | 0 | 0 | 0 | 1 | 0 | 0 |
| (J03.9) Acute tonsillitis, unspecified | 0 | 1 | 0 | 0 | 0 | 0 | 0 | 2 | 0 | 0 |
| (J10) influenza due to identified seasonal influenza virus | 0 | 10 | 0 | 0 | 0 | 0 | 0 | 0 | 0 | 0 |
| (J20.9) Acute bronchitis, unspecified | 0 | 0 | 0 | 0 | 0 | 0 | 0 | 1 | 0 | 0 |
| (J22) Unspecified acute lower respiratory infection | 0 | 2 | 0 | 1 | 1 | 3 | 0 | 0 | 0 | 3 |
| (R05) Cough | 0 | 0 | 0 | 0 | 0 | 0 | 0 | 0 | 1 | 0 |
| (R05.9) Cough, unspecified | 0 | 0 | 0 | 0 | 0 | 0 | 0 | 0 | 1 | 0 |
| (R09.81) Nasal Congestion | 0 | 0 | 0 | 0 | 0 | 0 | 0 | 0 | 2 | 0 |
| (U07.1) COVID-19, virus identified | 0 | 0 | 0 | 0 | 0 | 1 | 0 | 3 | 1 | 1 |
| (U07.2) COVID-19, virus not identified | 12 | 0 | 23 | 0 | 0 | 0 | 0 | 0 | 0 | 0 |
| Respiratory symptomps not classified (i.e., sore throat and head ache; dry cough and chilla) | 0 | 0 | 0 | 0 | 0 | 0 | 0 | 2 | 0 | 0 |
| **Total** | **12** | **27** | **23** | **5** | **7** | **10** | **0** | **15** | **13** | **8** |

Table S7. Medical codes for gastrointestinal syndromes of potential viral infectious origin recorded during the study period. Numbers indicate the cases reported in medical consultation report for the 7–10 days preceding arrival at the port

| Medical codes identified in the surveillance period for gastrointestinal symptoms | Company type and date of monitoring during 2024 | | | | | | | | | |
| --- | --- | --- | --- | --- | --- | --- | --- | --- | --- | --- |
|  | C, 15/07 | A, 22/07 | C, 30/07 | A, 07/08 | A, 26/08 | A, 09/09 | B, 16/09 | B, 02/10 | B, 14/10 | A, 14/10 |
| (A09.0) Other and unspecified gastroenteritis and colitis of infectious origin | 7 | 9 | 0 | 0 | 1 | 2 | 0 | 0 | 0 | 0 |
| (K29.0) Acute haemorrhagic gastritis | 0 | 0 | 0 | 0 | 0 | 0 | 0 | 0 | 1 | 0 |
| (K29.7) Gastritis, unspecified | 0 | 0 | 0 | 0 | 1 | 0 | 0 | 0 | 0 | 0 |
| (R10.3) Pain localized to other parts of lower abdomen | 0 | 1 | 0 | 1 | 1 | 1 | 0 | 0 | 0 | 1 |
| (R10.4) Other and unspecified abdominal pain | 0 | 0 | 0 | 0 | 0 | 1 | 0 | 0 | 0 | 0 |
| (R11) Nausea and vomiting | 0 | 2 | 0 | 1 | 3 | 2 | 0 | 0 | 1 | 0 |
| (R19.7) Diarrhea, unspecified | 0 | 0 | 0 | 0 | 0 | 0 | 0 | 3 | 2 | 0 |
| **Total** | **7** | **12** | **0** | **2** | **6** | **6** | **0** | **3** | **4** | **1** |
